# BoardION: real-time monitoring of Oxford Nanopore sequencing instruments

**DOI:** 10.1186/s12859-021-04161-0

**Published:** 2021-05-13

**Authors:** Aimeric Bruno, Jean-Marc Aury, Stefan Engelen

**Affiliations:** Génomique Métabolique, Genoscope, Institut François Jacob, CEA, CNRS, Univ Evry, Université Paris-Saclay, 91057 Evry, France

**Keywords:** Nanopore sequencing, Real-time monitoring, Web application

## Abstract

**Background:**

One of the main advantages of the Oxford Nanopore Technology (ONT) is the possibility of real-time sequencing. This gives access to information during the experiment and allows either to control the sequencing or to stop the sequencing once the results have been obtained. However, the ONT sequencing interface is not sufficient to explore the quality of sequencing data in depth and existing quality control tools do not take full advantage of real-time data streaming.

**Results:**

Herein, we present BoardION, an interactive web application to analyze the efficiency of ONT sequencing runs. The interactive interface of BoardION allows users to easily explore sequencing metrics and optimize the quantity and the quality of the data generated during the experiment. It also enables the comparison of multiple flowcells to assess library preparation protocols or the quality of input samples.

**Conclusion:**

BoardION is dedicated to people who manage ONT sequencing instruments and allows them to remotely and in real time monitor their experiments and compare multiple sequencing runs. Source code, a Docker image and a demo version are available at http://www.genoscope.cns.fr/boardion/.

## Background

Since 2014, Oxford Nanopore Technologies (ONT) has launched a range of nanopore sequencing devices, from the portable MinION device to the flexible, high-throughput PromethION sequencer. The ONT technology allows the sequencing of long DNA or RNA molecules with a read accuracy of currently 96% [1] and already has a wide range of applications [2–7]. Unlike traditional next-generation sequencing platforms, which provide data at the end of the sequencing experiment, ONT sequencing data is generated in real time. Users can access time-critical information [8] and thus be able to stop an analysis once the result is obtained. This eventually allows washing and reusing of the flowcells [9]. However, the ONT interface (MinKnow) does not provide enough interactivity and reporting to thoroughly explore the quality of the sequencing data. In addition, almost all quality control tools such as MinIONQC [10], pycoQC [11], NanoPack [12] and ToulligQC [13] do not take full advantage of the real-time capability of the ONT instrument as they analyze the sequencing data at the end of the run. Herein, we introduce BoardION, an interactive web application for real-time evaluation of ONT sequencing runs. BoardION offers the possibility for sequencing platforms to remotely and simultaneously monitor all their ONT devices (MinION, Mk1C, GridION and PromethION). It also allows the comparison of several sequencing experiments to assess the library’s preparations or the quality of the input samples. In addition, we compare BoardION and Minotour [14] which is currently the only software able to process the data in real-time.

## Implementation

BoardION is organized in two components, the first one is dedicated to the preprocessing of the files generated by the ONT basecaller (guppy) and the second one is a dynamic web application, the central part of BoardION, which allows the visualization and comparison of sequencing metrics. BoardION can be installed and used directly from the source code or via a docker image containing all the necessary dependencies. Documentation, github and dockerhub repositories, as well as an interactive demo version of BoardION are available at http://www.genoscope.cns.fr/boardion/.

### Data preprocessing

Assessment of an in-progress run is performed by using information contained in the sequencing summary file generated by the guppy basecaller, but this file is not directly read and load by the web application. Indeed, the sequencing summary can be quite large (more than 10 GB) and will become larger in the future with the increase of the throughput. Instead, a preprocessing tool analyzes it periodically to generate lightweight datasets that contain only the metrics of interest. On each launch, the software searches for the sequencing summary files in the input folder and uses the presence of the final_summary.txt file to detect completed experiments. This preprocessing step, developed in C++, uses the seekg function of the iostream library to directly access and process only newlines. With these optimizations, statistics can be computed and updated in the web application every minute. For each flowcell, the preprocessing script generates five small files (< 3 MB): four containing statistics calculated every 10 min and one containing the read length distribution. The C++ software also creates a file listing all the flowcells, their status (completed or not) and the latest metrics. The cpu time and memory usage of the preprocessing script are correlated with the number of reads of a run. It takes one minute and 60 Mo on average to process a single run and less than 5 min and 100 Mo on our largest PromethION run (83 M reads).

### BoardION web application

We developed a web application because it has the advantage of being easily accessible regardless of the user's configuration. Thus, users can remotely and simultaneously monitor all the sequencing runs of their ONT devices. The BoardION web application is based on the R shiny package [15] that allows us to describe the content of the interface and the relationships between the elements of the application. It also uses ggplot2 [16] and plotly R library [17] to create dynamic JavaScript graphs. The memory usage of the web application is dependent on the use of the interface, but generally requires less than 400 Mo. The BoardION application computes widely used metrics such as yield, translocation speed, read length and quality. All graphs, dynamically generated, can be exported and saved as images. The interface is divided into three tabs: «Run in progress» that emphasizes the sequencing experiments currently in-progress, «Run archive» that allows users to accurately evaluate a given experiment across all stored experiments and «Sequencing overview» that compares metrics from all sequencing experiments (in progress or finished).

**Fig. 1:**
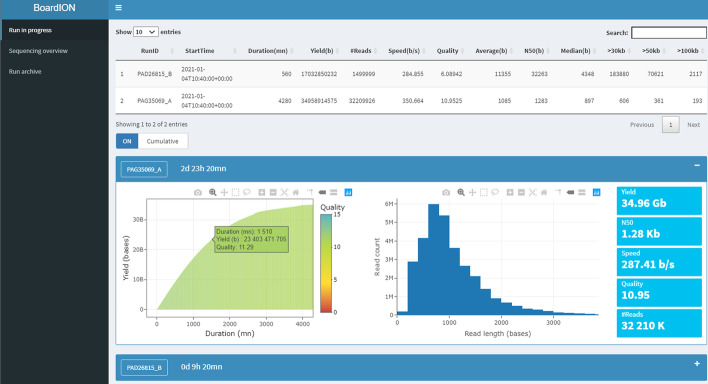
BoardION «Run in progress» tab. A first panel lists sequencing metrics of all in-progress experiments. Below, each panel is dedicated to one of these experiments. Users can open the panels to display the throughput, the read length distribution and important metrics of each experiment. The throughput can be plotted cumulatively from the start of the run or every ten minutes. All graphs are interactive as users can also zoom or display popup above the mouse to highlight particular values. In the example, the panel of the first experiment is opened while the second is minimized

The «Run in progress» tab displays an overview of the main sequencing metrics (read length, yield, quality, translocation speed …) in real time for all runs being sequenced (Fig. 1). Metrics are plotted cumulatively from the start of the experiment to assess the overall efficiency or every ten minutes to quickly detect a drop in quality. This dashboard allows users to follow the in-progress runs and to investigate more precisely a given experiment using the « Run archive» tab.

**Fig. 2:**
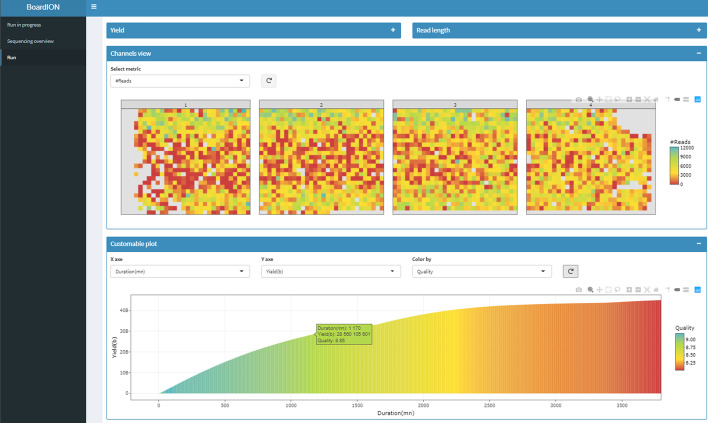
BoardION «Run archive» tab. Channel view panel: distribution on the flowcell channel grid of a metric chosen by the user. Customizable plot panel: the variation of the chosen metric is represented as a function of the elapsed time and colored according to the quality. In this example, yield and read length distribution panels are minimized

The «Run archive» tab contains the results of in-progress and finished runs (Fig. 2). Metrics are displayed through dynamic and customizable graphs as users can select the metrics to plot. These metrics can be observed temporally to analyze the run quality according to experiment time or spatially on the channel grid of the flowcell, to detect the presence of bubbles or contaminants, which can reduce the efficiency of the sequencing experiment.

**Fig. 3:**
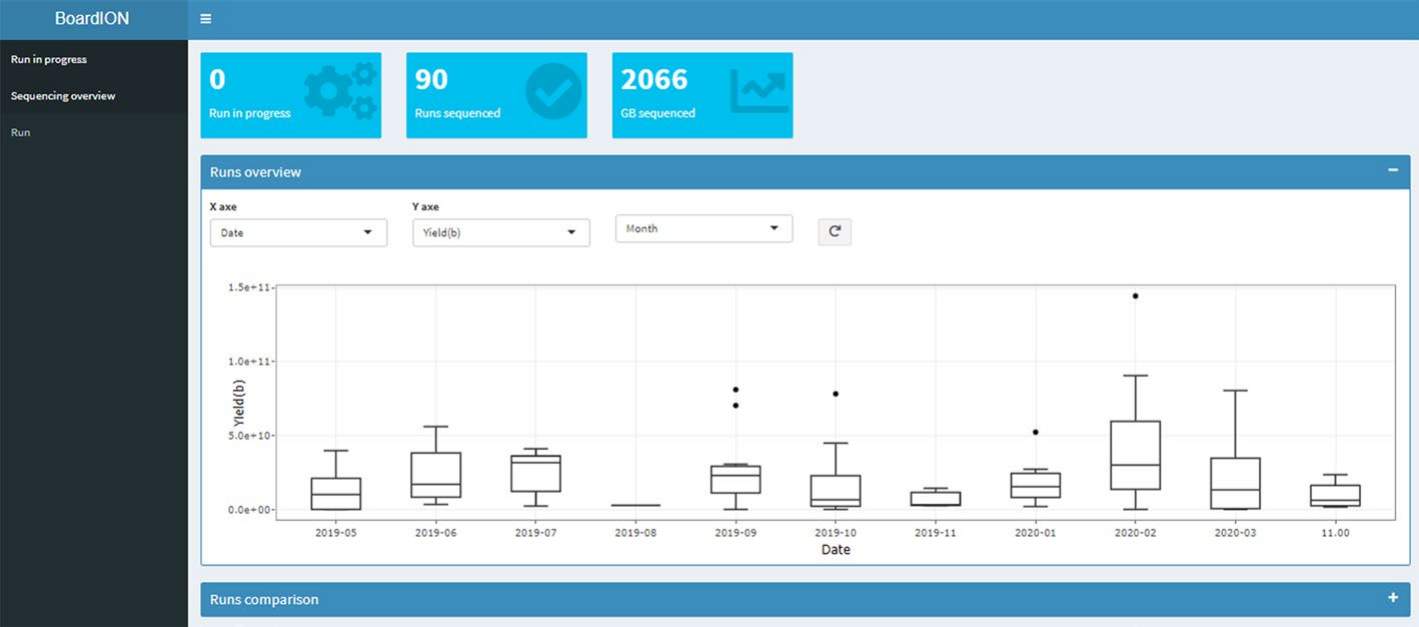
BoardION «Sequencing overview» tab. Run overview panel in which users can select run metrics to compare according to the run date. Here, the panel shows the monthly variation of the run yields. In run comparison panel users can select several runs to compare read length distributions and all other metrics over run time

The « Sequencing overview» tab is dedicated to the comparison of sequencing metrics from multiple flowcells (Fig. 3). This tab is flexible and the user can interactively choose between stored flowcells and proposed metrics and add them to current plots. The first panel gives an overview of the sequencing metrics for all stored sequencing runs and allows users to follow the evolution of the ONT technology according to the releases of new protocols, pores, sequencers or basecallers. Next panels are dedicated to the comparison of multiple runs by following a sequencing metric (yield, read size, translocation speed …) over run time or depicting read size distribution of several runs.

## Results and discussion

### Real-time monitoring

**Fig. 4:**
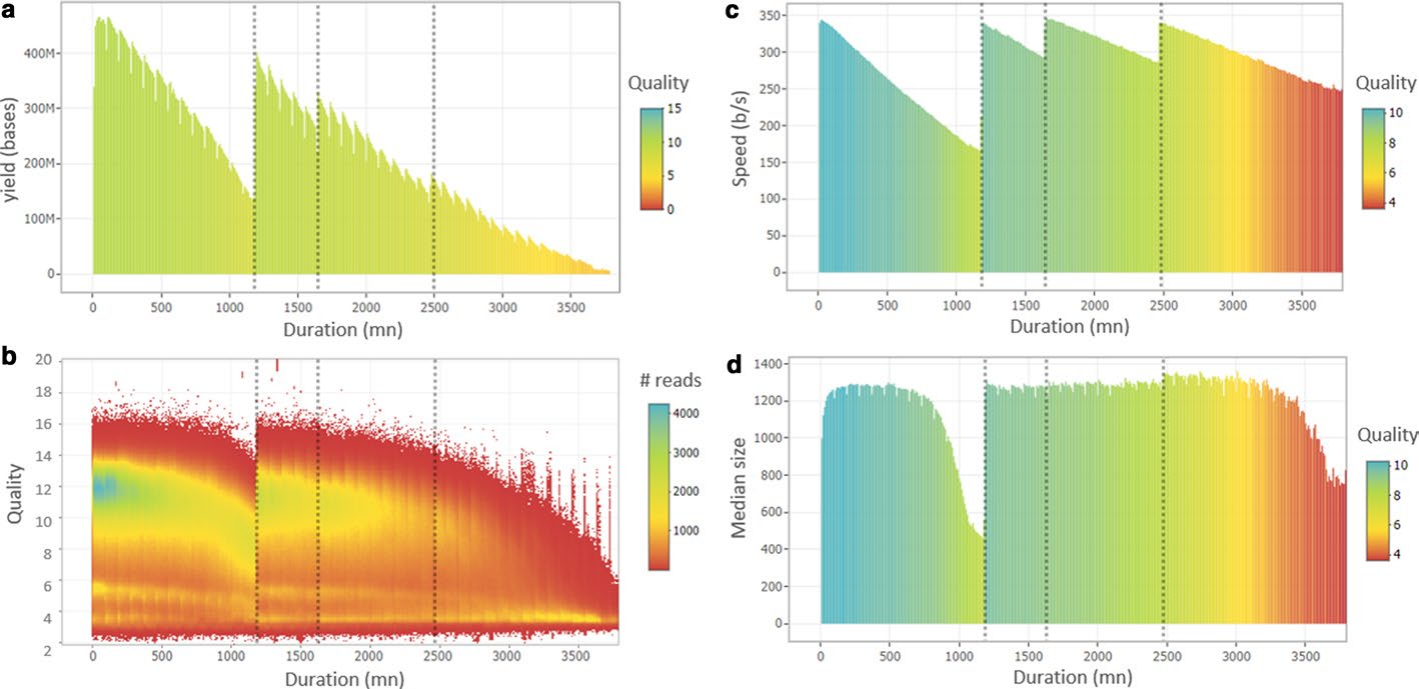
**a**, **c**, **d** Examples of customizable graphs for a refueled run: the metrics (yield, speed and median size) are calculated (sum or average) in ten minutes slices and displayed in function of the elapsed time. **b** Number of reads (color scale) of a given quality (y axis) generated during the sequencing experiment (x axis). Dotted black lines have been added manually and highlight the refueling time (1200, 1650 and 2450 mn). The plots show a drop of the median read size (**d**) and quality (**b**) after 500 mn of sequencing at a speed behind 260 b/s (**c**). The first refuel at 1200 mn restores initial speed (**c**) and median size (**d**) but also an almost initial yield per ten minutes (400 Mb vs. 460 Mb, plot **a**). The last two refuels were made when reaching a translocation rate around 300 b/s as recommended by ONT support

Generally, «Run in progress» and «Run archive» tabs are used to monitor ongoing sequencing experiments. During the sequencing, the consumption of reagents leads to a decrease in the translocation speed that can affect the read quality or the yield [1]. Indeed, ONT recommends keeping the translocation rate above 300 bases per second [18]. Thanks to its real-time capability, BoardION allows the users to detect a decrease of the translocation rate, and then add reagents (refueling) at the right time to avoid an irreversible drop in effectiveness. Figure 4 shows an example of a PromethION run, refueled after 20 h, following the observation of a decrease in translocation speed as well as read length and base quality. After refueling, the sequencing experiment recovers an optimal translocation rate, generates longer and higher-quality reads and finally achieves a yield of 83 Gb. In addition, BoardION allows sequencing platforms to define metric thresholds (speed, quality or yield) below which a flowcell must be refueled or washed to sequence a new sample.

### Comparison of flowcells

**Fig. 5:**
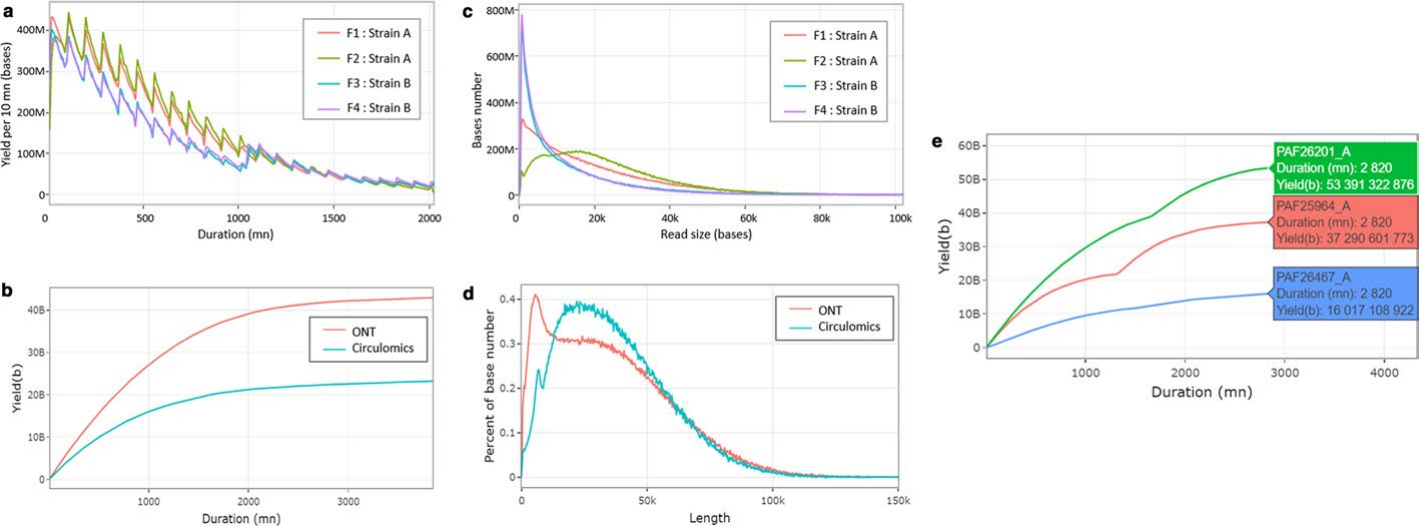
Examples of metrics and plots depicted by the run comparison panel of the sequencing overview tab. The panel allows us to compare runs according to yield and read length. **a** Ten minutes cumulative yield of four runs. **b** Cumulative yield from the sequencing start of two runs. **c** Number of bases contained in reads of a particular length of four runs. **d** Percent of bases contained in reads of a particular length of two runs. **e** Cumulative yield from the experiment start of three runs being sequenced

We compare four flowcells, where two strains of the same species were sequenced using different library sizes. We observe that the yield is not the same between the two strains (Fig. 5a). In contrast, the yield is the same for a given strain even if the size of the library is different (Fig. 5c). Library preparation like flowcell F2 seems to be a better option for generating longer reads. One main advantage of the ONT technology is the ability to sequence very long DNA fragments (> 30 kb). As an illustration, we also used BoardION to compare long-reads library preparation protocols of ONT and Circulomics by sequencing the same genotype of a plant. With the Circulomics protocol the peak at a read size of 5 kb is almost removed (Fig. 5d). This protocol generates a higher proportion of bases in reads longer than 10 kb. Nevertheless, the ONT protocol generates almost twice more bases than the Circulomics protocol (Fig. 5b). Another usage of the run comparison panel is the possibility to compare in real time the yield and the read size distribution of simultaneous runs being sequenced (Fig. 5e).

### Discussion and future developments

Like BoardION, the ONT sequencing interface Minknow2 displays real-time information on translocation speed, yield, read length and quality but adds graphs on temperature, voltage, pores states, barcodes distribution and reference coverage. Nevertheless, the graphs lack interactivity to fully explore the quality of the run. In contrast, BoardION provides interactivity to plot all the metrics cumulatively from the start of the run, every ten minutes, according to the channel grid of the flowcells or to correlate the metrics using customizable panels. Furthermore, BoardION adds some functionalities such as storing statistics of all sequenced runs, the comparison of the metrics of these runs and the possibility to remotely and simultaneously monitor the runs of many ONT devices. Minknow is a great application to monitor a single run, but BoardION is dedicated to the monitoring and the comparison of all the runs performed in a given facility.

MinoTour is currently the only platform able to process the ONT sequencing data in real-time. The platform is composed of a database to store sequencing metrics and a web user interface. The interface displays metrics describing run efficiency (yield, read length and quality, rate, pore activity) and alignment to a reference genome (coverage). Users can also setup alerts to be informed when a threshold is reached. We tested minoTour to compare its functionalities with BoardION. Installation of minoTour on unix is quite hard for biologists without strong computer skills. MinoTour developers propose to upload data on their server but due to web transfer this solution does not allow a real time analysis of data and is problematic in term of confidentiality. In contrast, the docker image of BoardION is easy to install and platform independent. We tested minoTour using data provided by the minoTour platform. Unlike BoardION, minoTour offers the possibility to explore the signal of the runs and the coverage obtained on the sequenced genome. However, BoardION has some additional features such as comparison of runs, overview of all runs and customizable plots that allow users to thoroughly explore their sequencing experiments. Furthermore, we did not succeed to upload our own sequencing data on the minoTour server. It seems that minoTour has not been updated since 2017, so, as ONT technology evolves quickly, minoTour seems to be currently unusable on recent ONT sequencing data.

Several applications can take advantages of all ONT generated data (assembly, structural variation) but others need only high quality data (identification, SNV, epigenetics). Guppy basecalling report distinguishes pass and fail reads (quality threshold of 7). It will be useful to report metrics for pass reads in order to help sequencing platforms to optimize the pass yield. It will also be useful to report the yield of each sample of barcoding runs to assess barcoding efficiency and evaluate the generated data obtained for each sample. We aim to add an alert feature that sends emails to users when a threshold is reached for a metric. With this feature, the sequencing platforms will be able to configure their own alert thresholds in order to react faster in case of sequencing problems or to stop a run once a yield is obtained. We also want to add a report generator to export readable documents containing all metrics and graphs computed by BoardION.

## Conclusion

BoardION is an interactive web application for real-time monitoring of ONT sequencing runs. The application can be installed via the Docker distribution. BoardION’s dynamic and interactive interface allows users to explore sequencing metrics easily and to optimize in real time the quantity and the quality of the generated data. It also offers the possibility of comparing several sequencing experiments, which can be useful to analyze sequencing conditions and follow the evolution of the ONT technology. We believe BoardION will help sequencing platforms to establish the best sequencing guidelines for different types of samples.

### Availability and requirements

Project name: BoardION.

Project home page: https://github.com/institut-de-genomique/BoardION.

Operating system(s): Platform independent.

Programming language: c++, R.

Other requirements: R library shiny 1.4.0.2, plotly 4.9.2.1 and ggplot2 3.3.1.

License: CeCILL.

Any restrictions to use by non-academics: no.

## Data Availability

Not applicable.

## Abbreviations

ONT: Oxford Nanopore Technology
DNA: Deoxyribo nucleic acid
GB: Giga bytes
MB: Mega bytes
SNV: Single nucleotid variation
